# Validity Examination of the Dissipative Quantum Model of Olfaction

**DOI:** 10.1038/s41598-017-04846-8

**Published:** 2017-06-30

**Authors:** Arash Tirandaz, Farhad Taher Ghahramani, Vahid Salari

**Affiliations:** 1 School of Biological Sciences, Institute for Research in Fundamental Sciences (IPM), P.O. Box 19395-5531 Tehran, Iran; 2 School of Physics, Institute for Research in Fundamental Sciences (IPM), P.O. Box 19395-5531 Tehran, Iran; 3 Department of Physics, Isfahan University of Technology, Isfahan, P.O. Box 84156-83111 Iran

## Abstract

Despite some inconclusive experimental evidences for the vibrational model of olfaction, the validity of the model has not been examined yet and therefore it suffers from the lack of conclusive experimental support. Here, we generalize the model and propose a numerical analysis of the dissipative odorant-mediated inelastic electron tunneling mechanism of olfaction, to be used as a potential examination in experiments. Our analysis gives several predictions on the model such as efficiency of elastic and inelastic tunneling of electrons through odorants, sensitivity thresholds in terms of temperature and pressure, isotopic effect on sensitivity, and the chiral recognition for discrimination between the similar and different scents. Our predictions should yield new knowledge to design new experimental protocols for testing the validity of the model.

## Introduction

**Figure 1:**
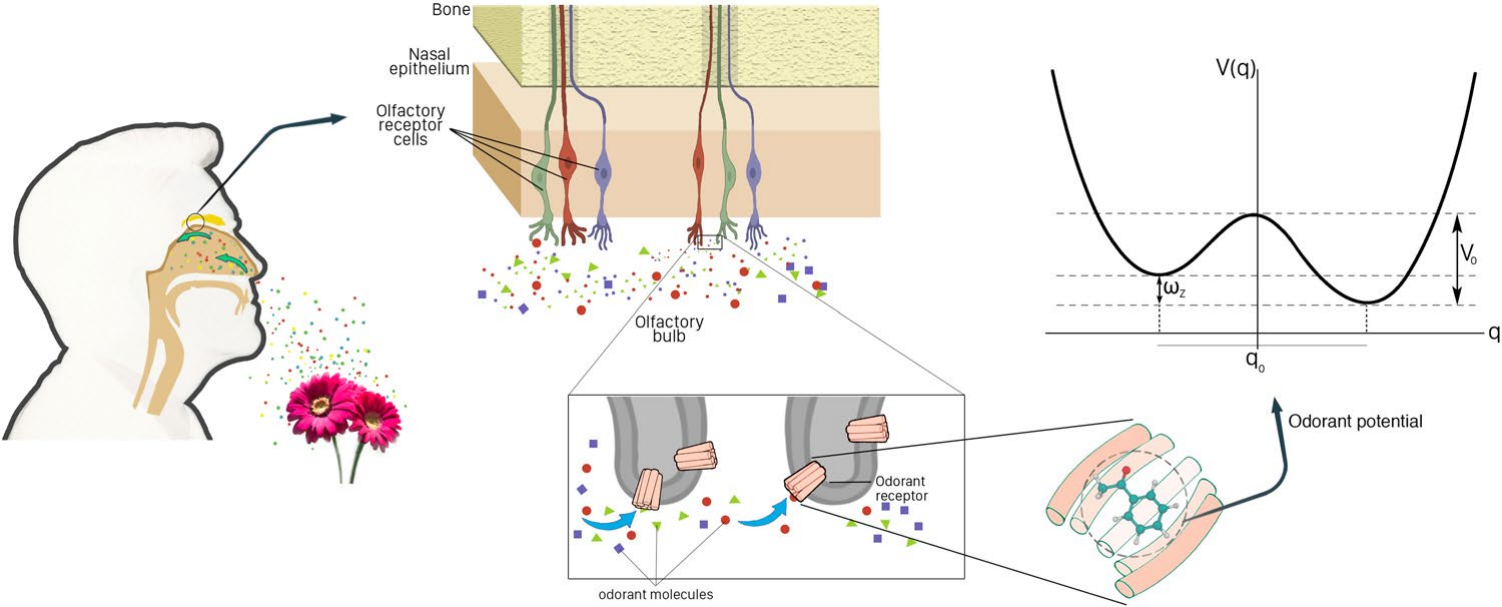
A scheme for the sense of smell in which odorants are absorbed by odorant receptors (ORs) in the olfactory receptor cells in the nasal cavity. In the quantum model, each odorant can be simulated as an asymmetric double-well potential for odorant recognition. The signal transduction relies on the success of an electron tunneling from a donor site of an OR to an acceptor site of the same or another OR, facilitated by a vibrational transition in the odorant according to the energy difference between the donor and acceptor sites.

Olfaction seems to be an immediate and intimate sense but surprisingly the mechanism is still not well understood. This is an important problem in its own right for both fundamental science and industry^1–5^. The olfactory system in human beings is triggered by binding the small, neutral, and volatile molecules known as odorants to specific sites on olfactory receptors (ORs) in the nasal cavity (see Fig. 1). Despite considerable knowledge of structure of ORs, the detailed molecular mechanisms for discrimination between different odorants are not yet fully understood^6^. In 1963, Amoore conjectured that such molecular mechanism is primarily related to the *shape* of the odorant and accordingly it is initiated by a mutual structural fit between the odorant and ORs (i.e. *lock and key* model)^7^. In fact, the idea was motivated from the molecular mechanism of the enzyme behaviour. The model can be modified by introducing a distortion of the whole system to induce a more appropriate mutual fit (i.e. *hand and glove* model). A more refined demonstration of the idea requires that ORs respond to only one structural feature, such as a functional group, instead the main body of the odorant (i.e. *odotope* model)^8^. There is plenty of evidence for cases where the structure does seem important to an odorant’s detection (e.g. see refs 9 and 10). Despite the predictive power of these structure-based models, there are some evidence against them: odorants that smell similarly whilst being structurally different (e.g. benzaldehyde versus hydrogen cyanide), and odorants that smell differently whilst being structurally the same (e.g. ferrocene versus nickelocene)^11–13^. All such shape-based models are primarily based on mechanical mechanisms.

The quantum model of olfaction, which was firstly proposed by Dyson^14^ and refined by Wright^15^, is based on the idea that the signature of scent is due to the odorant’s unique vibrational spectrum not its structure.

Motivated by the phenomena of inelastic electron tunneling (ET) in metals^16, 17^, Turin proposed that the mechanism of olfactory detection is an odorant-mediated biological inelastic ET^18^. The signal transduction relies on the success of an ET from a donor (D) site of an OR to an acceptor (A) site of the same or another OR, facilitated by a vibrational transition in the odorant corresponding to the energy difference between D and A sites. Recently, Brookes and co-workers formulated the idea in a semi-classical model to show that such a mechanism fits the observed features of smell^19^. They found that the rate of odorant-mediated inelastic ET and the elastic ET can be drastically different and surprisingly the inelastic ET is the dominant process for certain parameters of the model. The evidence supporting the vibration-based mechanism was obtained from sophisticated quantum chemistry calculations^20, 21^. Chȩcińska and co-workers examined the dissipative role of the environment dynamically in vibration-based model and showed that the strong coupling to the environment can enhance the frequency resolution of the olfactory system^22^. The main evidence against this theory is given to be the differentiable smells of chiral odorants: they have identical spectra in an achiral solution, but they have different smells^12^. Recently, we addressed this issue by using the master equation approach^23^ in which we used the fact that the ‘contortional’ vibration of the odorant describes the inter-conversion between its chiral states^24^. Such a vibration is described by a double-well potential. In this context, we showed that the olfactory chiral recognition can be realized by the recognition of the energy difference between chiral states. Even so, the ‘contortional’ vibration, and so does the double-well potential, is not restricted to chiral molecules. All non-planer molecules, chiral and achiral, have a contortional degree of freedom. A major prediction of the vibration-based theory is the isotope effect: i.e. isotopes should smell differently. Recent behavioural experiments have revealed that fruit flies^21, 25^, honeybees^26^ and humans^27, 28^ can distinguish isotopes. Yet, experimental evidence against isotopic discrimination keeps the debate open^29, 30^. Also the effect of pressure on the olfactory recognition is studied in experiment (see e.g. refs 31 and 32). However, these are controversial claims and there is no agreement on the actual effect. The theoretical analysis of pressure dependency is complicated. This is primarily because of the fact that the changes in the pressure of the odorant affects the bath spectrum itself.

In this paper, we examine the physical plausibility of the odorant-mediated inelastic ET model of olfaction. For the odorant, we focus on the contorsional vibration, in which an atom or a group of atoms oscillates between the two wells of the potential energy surface. The biological environment is conveniently represented as a collection of harmonic oscillators. We examine the dynamics of the odorant by using the time-dependent perturbation theory and thereby obtain the corresponding elastic and inelastic ET rates for all possible transitions of the odorant. To test the physical limitations of the model, we analyze the rates in different limits of molecular and environmental variables. For simplicity we set *ħ* = 1 throughout the paper.

## Model

**Figure 2:**
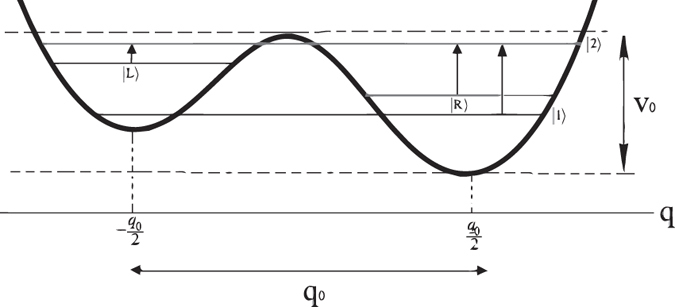
Possible transitions of the odorant described as a particle in an asymmetric double-well potential.

We focus on three parts of the system as the main components of the olfaction model: (1) the *odorant*, (2) the *electron* which tunnels through the odorant, and (3) the surrounding *environment*. In the original vibrational model of olfaction, the relevant vibrational mode is represented by a simple harmonic oscillator^19^. Here, we consider a more realistic vibrational mode of non-planer odorant, known as *contorsional* mode, in which an atom or a group of atoms oscillates between the left and right wells of a double-well potential. Unlike the harmonic mode the contorsional mode can be used to charachterize the olfactory chiral reconition^23^. Thus, we model the odorant as an asymmetric double-well potential (see Fig. 1). The minima of the potential correspond to the left- and right-handed states, |*L*〉 and |*R*〉, of the odorant (see Fig. 2). The handed states can be inter-converted by the quantum tunneling through the barrier *V*_0_. In the limit ${V}_{0}\gg {\omega }_{0}\gg {k}_{B}T$ (*ω*_0_ is the vibration frequency at the bottom of each well), the state space of the odorant is effectively confined in a two-dimensional Hilbert space spanned by two handed states. Such an approximation works properly for a large class of odorants even in the high-temperature limit^24, 33^. The odorant’s Hamiltonian can then be expanded by the handed states as ${\hat{H}}_{od}=-{\omega }_{z}{\hat{\sigma }}_{z}+{\omega }_{x}{\hat{\sigma }}_{x}$ where ${\hat{\sigma }}_{i}$ is the *i*-component of Pauli operator and *ω*_*x*_ and *ω*_*z*_ are the tunneling and asymmetry frequencies, respectively. The tunneling frequency *ω*_*x*_ can be calculated from the WKB method as ${\omega }_{x}=A{q}_{0}\sqrt{M{\omega }_{0}}\,\exp \,(-B{V}_{0}/{\omega }_{0})$ ^34^, where *M* is the molecular mass and *q*_0_ is the distance between two minima of the potential. The value of the parameters *A* and *B* depends on the explicit mathematical form of the potential, but it can usually be approximated by 1^34, 35^. The asymmetry is due to the fundamental parity-violating interactions^36, 37^ and the chiral interactions (i.e. interactions that are transformed as pseudoscalars^38^) between the odorant and environmental molecules. The former is typically small but the latter can be significant especially between a chiral odorant and ORs. The eigenstates of the odorant’s Hamiltonian can be written as the superposition of handed states as $|{E}_{1}\rangle =\,\sin \,\theta |L\rangle +\,\cos \,\theta |R\rangle$ and $|{E}_{2}\rangle =\,\cos \,\theta |L\rangle \,-\,\sin \,\theta |R\rangle$ where we defined $\theta =\mathrm{(1/2)}\,\arctan ({\omega }_{x}/{\omega }_{z})$.

The electron tunnels through the odorant from a donor state |*D*〉 with energy *ε*_*D*_ to an acceptor state *ε*_*A*_ with energy *ε*_*A*_. We then describe the electron with Hamiltonian ${\hat{H}}_{e}={\varepsilon }_{A}|A\rangle \langle A|+{\varepsilon }_{D}|D\rangle \langle D|$. The biological environment is typically modeled as a collection of harmonic oscillators with Hamiltonian ${\hat{H}}_{env}={\sum }_{i}\,{\omega }_{i}{\hat{b}}_{i}^{\dagger }{\hat{b}}_{i}$ where ${b}_{i}^{\dagger }$ and *b*_*i*_ are the creation and annihilation operators for modes of frequency *ω*_*i*_ in the environment.

The interaction Hamiltonian has three contributions: between donor and acceptor of the receptor with tunneling strength Δ, between the donor (acceptor) and the odorant with coupling frequency *γ*_*D*_ (*γ*_*A*_), and between the donor (acceptor) sites and *i*-the harmonic oscillator of the environment with coupling frequency *γ*_*iD*_ (*γ*_*iA*_). Thus, the interaction Hamiltonian of the whole system is given by ref. 19

1 $$\begin{array}{rcl}{\hat{H}}_{int} & = & {\rm{\Delta }}(|A\rangle \langle A|+|D\rangle \langle D|)+({\gamma }_{D}|D\rangle \langle D|+{\gamma }_{A}|A\rangle \langle A|)\otimes {\hat{\sigma }}_{x}\\  &  & +\sum _{i}({\gamma }_{i,D}|D\rangle \langle D|+{\gamma }_{i,A}|A\rangle \langle A|)\otimes ({\hat{b}}_{i}^{\dagger }+{\hat{b}}_{i})\end{array}$$

The total Hamiltonian characterizes the time evolution of whole system by which the ET rates are calculated. The details of the dynamics are explained in the Methods section.

## Results

### Tunneling Rates

When the electron tunnels through the odorant, three type of transitions might take place in the odorant (Fig. 2): $|L\rangle \to |{E}_{2}\rangle$, $|R\rangle \to |{E}_{2}\rangle$ and $|{E}_{1}\rangle \to |{E}_{2}\rangle$.

The ET rates are obtained from the corresponding probabilities (see Methods) by using ${{\rm{\Gamma }}}_{i\to j}=dP{r}_{i\to j}/dt$. If we set *t*_1_ = 0, in the high-temperature limit where the environment is presumably in thermal equilibrium, the inelastic ET rates are given by

2 $$\begin{array}{rcl}{{\rm{\Gamma }}}_{D,L\to A,{E}_{2}} & = & {{\rm{\Delta }}}^{2}\sqrt{\tfrac{\pi }{{k}_{B}T{J}_{0}\lambda }}\,\cos \,(\theta +\upsilon )\,[\cos \,\theta \,\cos \,\upsilon \,\exp \,\{\tfrac{-{(\epsilon -{J}_{0}\lambda +({\eta }_{D}-{\eta }_{A}))}^{2}}{4{k}_{B}T{J}_{0}\lambda }\}\\  &  & -\,\sin \,\theta \,\sin \,\upsilon \,\exp \,\{\tfrac{-(\epsilon -{J}_{0}\lambda -({\eta }_{A}+{\eta }_{D}))}{4{k}_{B}T{J}_{0}\lambda }\}]\end{array}$$

3 $$\begin{array}{rcl}{{\rm{\Gamma }}}_{D,R\to A,{E}_{2}} & = & {{\rm{\Delta }}}^{2}\sqrt{\tfrac{\pi }{{k}_{B}T{J}_{0}\lambda }}\,\sin \,(\theta +\upsilon )\,[\sin \,\theta \,\cos \,\upsilon \,\exp \,\{\tfrac{-{(\epsilon -{J}_{0}\lambda -({\eta }_{D}-{\eta }_{A}))}^{2}}{4{k}_{B}T{J}_{0}\lambda }\}\\  &  & +\,\cos \,\theta \,\sin \,\upsilon \,\exp \,\{\tfrac{-{(\epsilon -{J}_{0}\lambda +({\eta }_{A}+{\eta }_{D}))}^{2}}{4{k}_{B}T{J}_{0}\lambda }\}]\end{array}$$

4 $$\begin{array}{rcl}{{\rm{\Gamma }}}_{D,{E}_{1}\to A,{E}_{2}} & = & {{\rm{\Delta }}}^{2}\sqrt{\frac{\pi }{{k}_{B}T{J}_{0}\lambda }}\{{\sin }^{2}\,(\upsilon )\,[{\cos }^{2}\,\theta \,\exp \,\{\frac{-{(\epsilon -{J}_{0}\lambda -({\eta }_{A}+{\eta }_{D}))}^{2}}{4{k}_{B}T{J}_{0}\lambda }\}\\  &  & +\,{\sin }^{2}\,\theta \,\exp \,\{\frac{-{(\epsilon -{J}_{0}\lambda +({\eta }_{A}+{\eta }_{D}))}^{2}}{4{k}_{B}T{J}_{0}\lambda }\}]\\  &  & +\,\frac{1}{4}\,\sin \,\theta \,\sin \,(\upsilon )\,[\exp \,\{\frac{-{(\epsilon -{J}_{0}\lambda -({\eta }_{D}-{\eta }_{A}))}^{2}}{4{k}_{B}T{J}_{0}\lambda }\}\\  &  & -\,\exp \,\{\frac{-{(\epsilon -{J}_{0}\lambda +({\eta }_{D}-{\eta }_{A}))}^{2}}{4{k}_{B}T{J}_{0}\lambda }\}]\}\end{array}$$

where $\upsilon =\frac{1}{2}[{\tan }^{-1}\,(\frac{{\omega }_{x}+{\gamma }_{A}}{{\omega }_{z}})+{\tan }^{-1}\,(\frac{{\omega }_{x}-{\gamma }_{D}}{{\omega }_{z}})]$. The elastic ET coincides with the situation where the electron tunnels from the donor site to the acceptor site, without any transition in the odorant. This situation can be considered to be equivalent to the ET in the absence of the odorant^22^. The elastic ET rate in the high-temperature limit is given by

5 $${{\rm{\Gamma }}}_{D\to A}={{\rm{\Delta }}}^{2}\sqrt{\frac{\pi }{{k}_{B}T{J}_{0}\lambda }}\,\exp \,\{\frac{-{(\epsilon -{J}_{0}\lambda )}^{2}}{4{k}_{B}T{J}_{0}\lambda }\}$$

### Physical Parameters

To examine the obtained ET rates quantitatively we first analyze the parameters of the model. Since we aim to examine the model in experiment, the ET rates should be analyzed in terms of controllable parameters (aka variables). These variables include odorant’s parameters (tunneling frequency *ω*_*x*_ and asymmetry frequency *ω*_*z*_), and thermodynamical parameters of the environment (temperature and pressure). The parameters with interaction character naturally depend on the odorant’s parameters. The energy conservation requires that the energy gap between the donor and acceptor sites *ε* be close to the mean value of energy gap between odorant’s states. Thus we assume that $\varepsilon \simeq \sqrt{{\omega }_{x}^{2}+{\omega }_{z}^{2}}$. The coupling between donor and acceptor sites of OR(s) is weak in comparison with the natural frequency of the odorant, so we estimate ${\rm{\Delta }}\simeq 0.01\sqrt{{\omega }_{x}^{2}+{\omega }_{z}^{2}}\,{\rm{Hz}}$ ^23^. The coupling frequency between the DA pair and odorant, calculated from the Huang-Rhys factor^19^, is approximated as ${\gamma }_{D}=-{\gamma }_{A}\approx 0.1\sqrt{{\omega }_{x}^{2}+{\omega }_{z}^{2}}$ ^23^. Since the biological environment is microscopically uncontrollable, the parameters of the corresponding spectral density is considered as mere parameters. A natural environment for the odorant consists of OR(s) and the surrounding solvent. The simplest model arises when the odorant is treated as a point dipole inside a uniform and spherical protein surrounded by a uniform polar solvent. For a Debye solvent and a protein with a static dielectric constant, the spectral density is Ohmic with Drude-form cut-off as refs 39 and 40

6 $$J(\omega )=\frac{\alpha \omega }{1+{\lambda }^{-2}{\omega }^{2}}$$

with

7 $$\alpha =\frac{{({\rm{\Delta }}\mu )}^{2}}{4\pi {\epsilon }_{\circ }{b}^{3}}\frac{6{\epsilon }_{p}({\epsilon }_{s}-{\epsilon }_{\infty })}{\mathrm{(2}{\epsilon }_{s}+{\epsilon }_{p})(2{\epsilon }_{\infty }+{\epsilon }_{p})\lambda }$$

and

8 $$\lambda =\frac{2{\epsilon }_{s}+{\epsilon }_{p}}{2{\epsilon }_{\infty }+{\epsilon }_{p}}{\lambda }_{D}$$

where *b* is the radius of the protein containing the odorant, Δ*μ* is the difference between the dipole moment of the odorant in the ground and excited states, ${\epsilon }_{p}$ is the dielectric constant of the protein environment, ${\epsilon }_{s}$ and ${\epsilon }_{\infty }$ are the static and high-frequency dielectric constants of the solvent, and *λ*_*D*_ is the Debye relaxation frequency of the solvent. For an odorant in the aqueous environment, we have ${J}_{0}\approx 1$ and $\lambda \approx {10}^{12}\,{\rm{Hz}}$. The choice of the cut-off does not change the structure of the environment and thus the exponential cut-off yields the same dynamics.

### Odorant Analysis

We examine the ET rates for different odorants in terms of their molecular parameters, *ω*_*x*_ and *ω*_*z*_. The magnitude of tunneling frequency *ω*_*x*_, ranging from the inverse of the lifetime of the universe to millions of hertz, can be extracted from the spectroscopic data^41^. The asymmetry frequency of the odorant, *ω*_*z*_, represents an overall measure of all chiral interactions involved. For our system, these interactions are primarily due to the intermolecular interactions between the odorant and ORs. The magnitude of intermolecular interactions can in principle be determined by using quantum chemistry calculations.

We used a polaron-transformed Hamiltonian to perturbatively calculate transition probabilities in the Methods section. The validity of this technique depends on the coupling frequency Δ being much smaller than the cut-off frequency of the environment *λ*. The papameter Δ itself depends on the odorant’s parameters. For odorants, being relatively large molecules, the maximum value of *ω*_*x*_ is about 10^12^ Hz (see Table 2 of ref. 41). Regarding the asymmetry frequency, *ω*_*z*_, to our knowledge, only the contribution of electro-weak parity-violating interactions is theoretically calculated (see Table 2 of ref. 41). Nonetheless, we can estimate its maximum value by the enthalpy of mixing of two enantiomrers of a chiral odorant, which is in the order of 10^12^ Hz^42^. As a consequence, the maximum value of Δ would be always two orders of magnitude smaller than *λ*.

**Figure 3:**
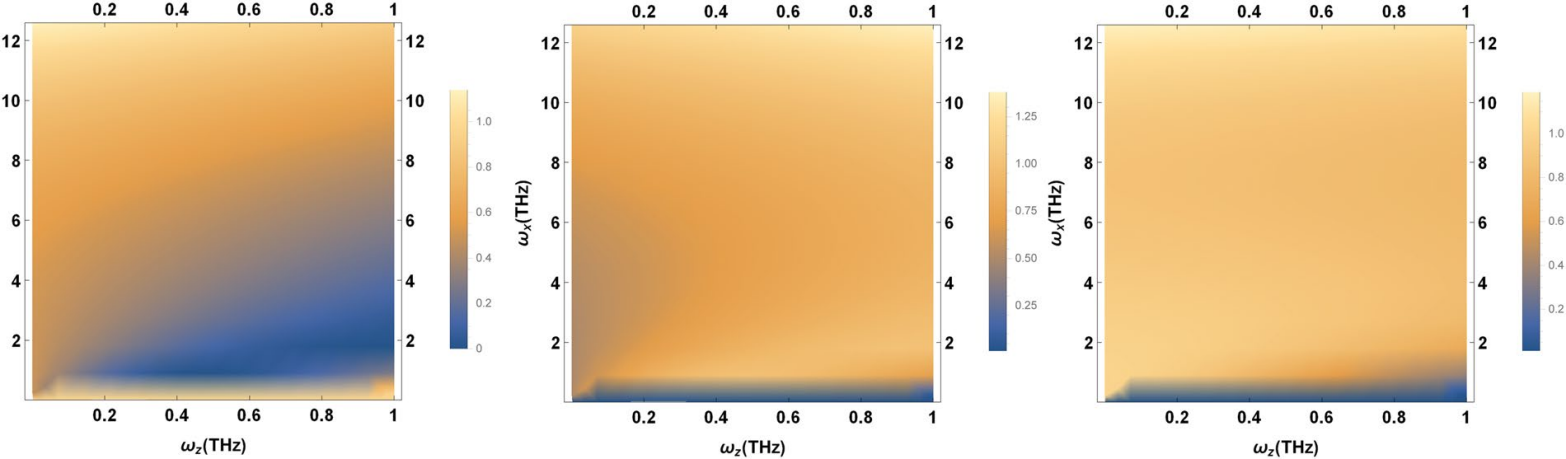
The inelastic-to-elastic ratio versus tunneling frequency *ω*_*x*_ and asymmetry frequency *ω*_*x*_ at biological temperature *T* = 310 *K* for transitions (Left) *L* → *E*_2_, (Middle) *R* → *E*_2_, and (Right) *E*_1_ → *E*_2_.

**Figure 4:**
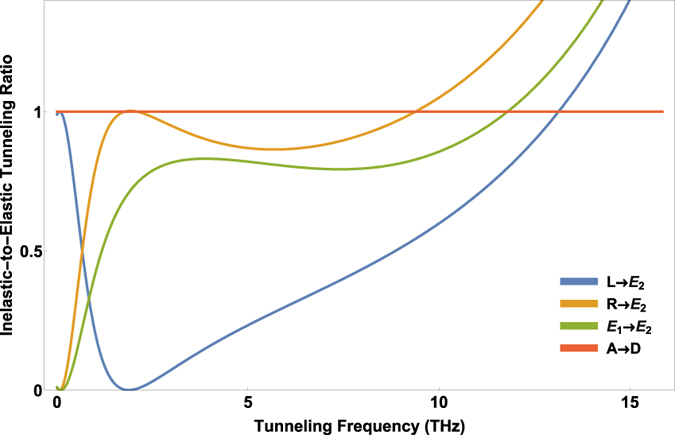
The inelastic-to-elastic ratio versus the tunneling frequency *ω*_*x*_ at biological temperature *T* = 310 *K* for transitions *L* → *E*_2_, *R* → *E*_2_ and *E*_1_ → *E*_2_. All figures are plotted at *ω*_*z*_ = 10^12^ Hz.

The dependence of the ET rates to *ω*_*x*_ and *ω*_*z*_ for different transitions of the odorant is plotted in Fig. 3. This shows that the vibrational model based on odorant-mediated inelastic ET is improbable for a wide range of odorants. Since ORs are chiral structures, we assume that the contribution of chiral interactions is significant. At a fixed high magnitude of asymmetry parameter *ω*_*z*_, the inelastic-to-elastic ratio against the tunneling frequency *ω*_*x*_ are plotted in Fig. 4 for different transitions of the odorant. Two different behaviors can be identified here; in the asymmetry-dominant limit, *ω*_*x*_ < *ω*_*z*_, although different transitions exhibit different dependencies on *ω*_*x*_, the inelastic ET is not dominant. In the tunneling-dominant limit, *ω*_*x*_ ≥ *ω*_*z*_, however, for all transitions the inelastic ET is dominant. In other words, for each transition there is a threshold of tunneling frequency *ω*_*x*_ below which the olfactory system struggles to recognize the odorant. This fact can be used to examine the model in experiment.

### Temperature Analysis

**Figure 5:**
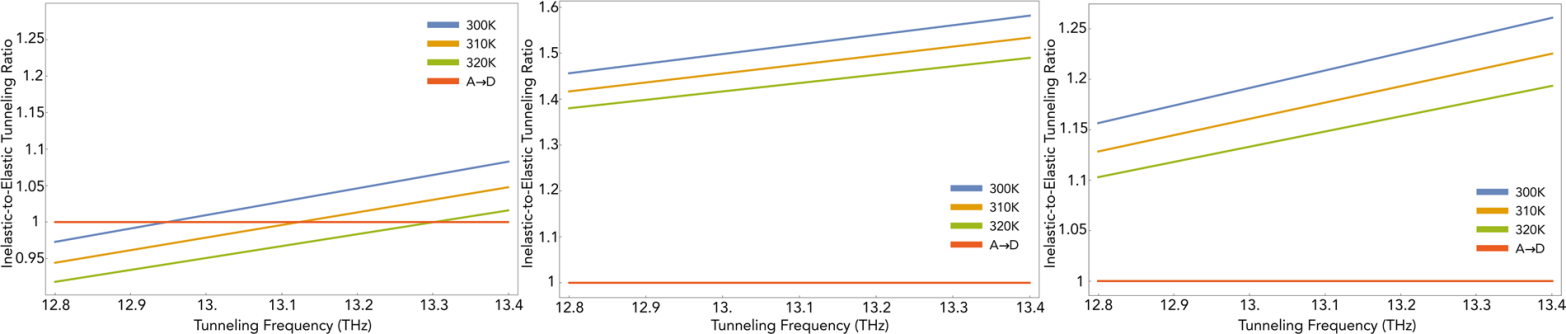
The inelastic-to-elastic ratio versus the tunneling frequency *ω*_*x*_ at temperatures *T* = 300 K, 310 K, and 320 K for transitions (Left) *L* → *E*_2_, (Middle) *R* → *E*_2_, and (Right) *E*_1_ → *E*_2_. All figures are plotted at *ω*_*z*_ = 10^12^ Hz.

The temperature dependency of different transitions of the odorant are essentially similar. At a fixed high magnitude of asymmetry parameter *ω*_*z*_, for the transitions *L* → *E*_2_, *R* → *E*_2_, and *E*_1_ → *E*_2_ the inelastic-to-elastic ratio versus the tunneling frequency *ω*_*x*_ are plotted in Fig. 5 for different temperatures of the environment. For each odorant (with a fixed tunneling frequency *ω*_*x*_) there is a threshold for temperature in the lower limit in which the olfactory system struggles to recognize the scent. This fact can also be used to examine the model in experiment.

### Pressure Analysis

The concentration of the odorant in the condensed environment is proportional to its pressure. The tunneling frequency of odorants is related to the pressure of the environment. To illustrate the pressure dependency of the odorant’s dynamics, we focus on the ammonia molecule *NH*_3_ as odorant. In the low-pressure limit, the tunneling frequency of ammonia, known as inversion frequency, is estimated as ${\omega }_{x}\simeq 2.4\times {10}^{10}\,{\rm{Hz}}$ ^43^, and at $P\simeq 2\,atm$, *ω*_*x*_ shifts to zero. This phenomenon is theoretically demonstrated in the context of the mean-field theory^44^. The pressure dependency of the inversion frequency is given by ${\omega }_{x}^{^{\prime} }={\omega }_{x}\sqrt{1-P/{P}_{cr}}$ where the critical pressure *P*_*cr*_ is approximately 1.6 *atm* at room temperature. If *P* → *P*_*cr*_, then *ω*′ → 0, and accordingly the handed states become eigenstates of the molecular Hamiltonian. In the high-pressure limit, the relevant transition in the odorant would be |*R*〉 → |*L*〉 (see Fig. 2). The inelastic ET rate according to this transition is obtained as

9 $${{\rm{\Gamma }}}_{D,R\to A,L}={{\rm{\Delta }}}^{2}\sqrt{\frac{\pi }{{k}_{B}T{J}_{0}\lambda }}\,{\sin }^{2}\,\upsilon \,\exp \,\{\frac{-{(\epsilon -{J}_{0}\lambda +({\eta }_{1}+{\eta }_{2}))}^{2}}{4{k}_{B}T{J}_{0}\lambda }\}$$

**Figure 6:**
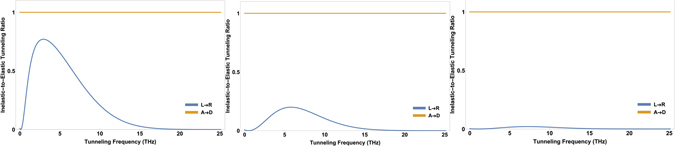
The inelastic-to-elastic ratio versus the tunneling frequency *ω*_*x*_ in the high-pressure limit for transition *DR* → *AL* for (Left) *ω*_*z*_ = 1 THz, (Middle) *ω*_*z*_ = 5 THz, and (Right) *ω*_*z*_ = 10 THz.

The inelastic-to elastic ratio versus the tunneling frequency *ω*_*x*_ in the high-pressure limit is plotted in Fig. 6 for *ω*_*z*_ = 1 THz, 5 THz, and 10 THz respectively.

In the high-pressure limit, the inelastic ET is always ineffective (see Fig. 6). This provides another empirical test for probing the validity of the tunneling model of olfaction. Specially, we predict that at *P* ≥ *P*_*cr*_ the olfactory system struggles to recognize the smell if the tunneling mechanism is at work.

Here, we assume that the spectral density of the environment is independent of the odorant’s concentration. To be more precise, we should examine how the parameters of the spectral density are affected by the change of the odorant’s concentration. Using the relations (7) and (8), one can easily show that the coupling frequency *J*_0_ and the cut-off frequency *λ* have an inverse and direct relation with the odorant’s concentration, respectively. Consequently, in the end, when we change the odorant’s concentration, the interplay between *J*_0_ and *λ* determines the change in *J*(*ω*).

### Isotopic Effect

The isotopic dependency of ET rates can be followed from the mathematical form of critical pressure as ${P}_{cr}=\sqrt{2{\omega }_{x}^{2}m{k}_{B}T}/\sigma (P)$ ^45^ where we defined $m=\mu {m}_{t}/(\mu +{m}_{t})$ with *μ* as the reduced mass of the odorant, *m*_*t*_ as a measure of average mass of the odorant and environment, and *σ*(*P*) is the decoherence cross-section. Using the hard sphere model for the odorant at room temperature, the decoherence cross-section can be approximated as 150*a*_*b*_ (*a*_*b*_ is the Bohr radius). Isotopic substitution alters *m* and its effect is significant for heavier odorants. Replacing an isotope with another with larger mass causes increasing *P*_*cr*_. According to ${\omega }_{x}^{^{\prime} }={\omega }_{x}\sqrt{1-P/{P}_{cr}}$ if *P*_*cr*_ is increased then ${\omega }_{x}^{^{\prime} }$ approaches to *ω*_*x*_ and we arrive the limit in which the inelastic ET is effective. As a result, the substitution of massive isotopes amplifies the sensitivity of possible quantum mechanism of odorant discrimination. For large odorants, the sensitivity is affected considerably. Exact values of *P*_*cr*_ can be calculated for different odorants using computational methods. So, we expect that the ability to distinguish a specific odorant is decreased when exposed to the pressures near *P*_*cr*_. Moreover, when we replace atoms of the odorant with their heavier isotopes, we predict that the same behavior appears in pressures larger than the *P*_*cr*_ of the original odorant.

### Chiral Recognition

The existence of enantiomers, which have different smells, are usually used as an argument to reject the quantum model of olfaction^21^. Since such enantiomers have the same vibrational spectrum, it seems that the shape-based parameters should be included in the quantum model to distinguish them from each other. The model presented here can be used to generalize the vibrational model for chiral recognition. Chiral molecules can be effectively modeled by a double-well potential^33^. The shape factor is the configuration of the chiral odorant. Our model predicts that the ET rates of inelastic ET are different for the two enantiomers for transitions |*L*〉 → |*E*_2_〉 and |*R*〉 → |*E*_2_〉 (see equations (2) and (3)). Our results here are in agreement with the Born-Markov master equation approach^23^. The values of elastic and inelastic ET rates are given in Table 1 for a series of odorant parameters. The enantiomers with similar smells lie in the limit ${\omega }_{x}\ll {\omega }_{z}$ (i.e. the first block in the Table 1). But the inelastic ET is ineffective in this limit. The enantiomers have different smells in the limit ${\omega }_{x}\approx {\omega }_{z}$ (i.e. the second block in the Table 1), but inelastic ET is still ineffective. The inelastic ET is effective for all transitions in the limit ${\omega }_{x} > {\omega }_{z}=\lambda$ (i.e. the third row of third block in the Table 1). In this limit, the ratio of the inelastic ET rate for the left-handed enantiomer to that of the right-handed one increases with the ratio of the tunneling frequency to the asymmetry frequency. Typical times for electron transfer in proteins are of order 10^−15^–10^−12^ *s* ^46^. Although the difference between inelastic ET rates of transitions may be appeared insignificant, however, in comparison with similar processes in biology it can be possible for the system to discriminate between two enantiomers under quantum constraints.

**Table 1:** Elastic and inelastic ET rates for some parameters of the chiral odorants at biological temperature *T* = 310 *K*.

*ω*_*x*_ Hz	*ω*_*x*_ Hz	${{\boldsymbol{\Gamma }}}_{{\boldsymbol{D}}{\boldsymbol{\to }}{\boldsymbol{A}}}^{-{\bf{1}}}\,{\bf{s}}$	${{\boldsymbol{\Gamma }}}_{{\boldsymbol{D}},{\boldsymbol{L}}{\boldsymbol{\to }}{\boldsymbol{A}},{{\boldsymbol{E}}}_{{\bf{2}}}}^{-{\bf{1}}}\,{\bf{s}}$	${{\boldsymbol{\Gamma }}}_{{\boldsymbol{D}},{\boldsymbol{R}}{\boldsymbol{\to }}{\boldsymbol{A}},{{\boldsymbol{E}}}_{{\bf{2}}}}^{-{\bf{1}}}\,{\bf{s}}$
10^3^	10^6^	15049.3	15195.3	$1.56632\times {10}^{6}$
10^6^	10^9^	0.0150482	0.0151942	1.56632
10^9^	10^12^	$1.44896\times {10}^{-8}$	$1.46302\times {10}^{-8}$	$1.56721\times {10}^{-6}$
10^12^	10^13^	$3.19822\times {10}^{-9}$	$3.20438\times {10}^{-9}$	$1.65938\times {10}^{-6}$
10^3^	10^3^	$7.52468\times {10}^{9}$	$3.68835\times {10}^{10}$	$9.45326\times {10}^{9}$
10^6^	10^6^	7524.68	36883.5	9453.26
10^9^	10^9^	0.00752388	0.0368776	0.00945288
10^12^	10^12^	$7.29208\times {10}^{-9}$	$3.5273\times {10}^{-8}$	$9.31282\times {10}^{-9}$
10^6^	10^3^	15049.3	30196.	30002.
10^9^	10^6^	0.0150482	0.0151942	1.56632
10^12^	10^9^	$1.44896\times {10}^{-8}$	$2.99804\times {10}^{-8}$	$2.97857\times {10}^{-8}$
10^13^	10^12^	$3.19822\times {10}^{-9}$	$3.69778\times {10}^{-10}$	$2.38954\times {10}^{-10}$

## Summary

Our analysis can be summarized as follows for the main ingredients of the olfactory system:

- *Odorant*: In the original vibrational model of olfaction, the relevant vibrational mode is represented by a simple harmonic oscillator^19^. Here, we focused on a more realistic vibrational mode of non-planer odorant, known as *contorsional* mode, in which an atom or a group of atoms oscillates between the left and right wells of a double-well potential. Unlike the harmonic mode the contorsional mode can be used to charachterize the olfactory chiral reconition^23^. The eigenstates of the double-well potential are essentially doplets. The first doplet is energetically available for most molecules at room temperature^24, 33^. The two-dimensional Hamiltonian of the mode is expressed by the tunneling frequency *ω*_*x*_ and asymmetry frequency *ω*_*z*_. Our expressions for the (in-)elastic ET rates are reduced to the corresponding expressions for a harmonic mode in the limit *ω*_*x*_ → 0^22^.
- *Electron*: The detailed biological origin of the electron which tunnels through the odorant is not known but it may be due to redox agents in the cell fluid^1^. According to the original model^19^, we considered donor (D) and acceptor (A) sites of traveling electron as single molecular orbitals with energies *ε*_*D*_ and *ε*_*D*_, coupled to each other by a weak hopping integral Δ. In order to satisfy energy conservation during the tunneling process, the electron’s parameters should be consistent with the odorant’s paramaters. Thus, they cannot be considered as variables.
- *Environment*: We modeled the biological environment as a harmonic bath with an ohmic spectral density according to the original model^19^. Such an environment is characterized by its microscopic parameters (e.g. coupling frequency *J*_0_ and cut-off frequency *λ*) and macroscopic parameters (e.g. temperature and pressure). Unlike the microscopic parameters, the macroscopic parameters can be controlled in experiment and thus they are considered as variables.

## Conclusion

In this paper, we have generalized and proposed an analysis for examination of the dissipative quantum model of olfaction in experiments. In fact, it has been suggested that inelastic ET through the odorant potential is a dominant process in olfaction. Here, we have suggested a region of easy measurable parameters in the lab (e.g. temperature and pressure) in which we can discriminate between the elastic and inelastic ET through the potential. We have shown that the dissipative odorant-mediated inelastic ET mechanism of olfaction is ineffective for a wide range of odorants, and the range of ineffectiveness depends on the type of transition in the contortional mode. Moreover, our results indicate that there are thresholds in the lower limit for both temperature and pressure in which the olfactory system cannot recognize the scent. Additionally, the substitution of massive isotopes amplifies the sensitivity of olfactory odorant discrimination. Perhaps the most relevant part of our analysis is related to the chiral recognition of the odorants in which enantiomers with similar smells lie in the asymmetry-dominant limit of dynamics and enantiomers with different smells lie in the tunneling-dominant limit of dynamics.

We expect that our analysis can be used in experiments to examine the validity of the dissipative quantum model of olfaction.

## Methods

### Initial States

**Figure 7:**
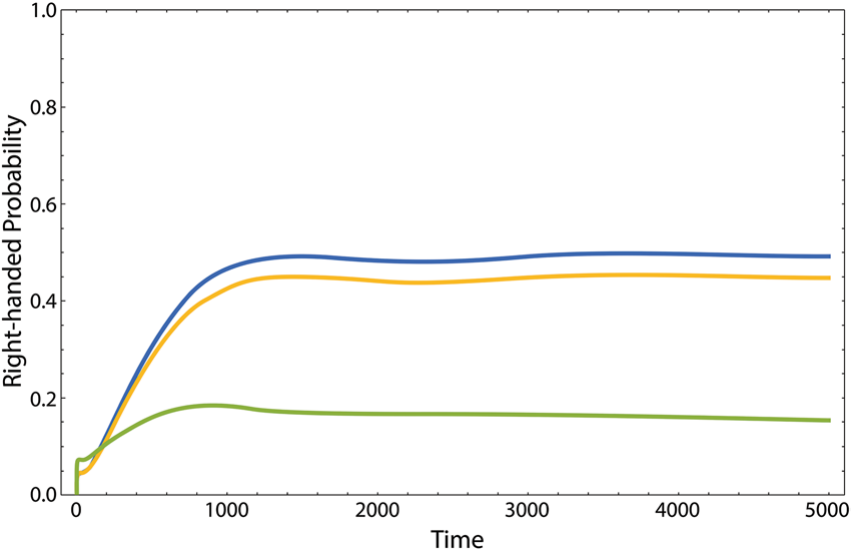
The dynamics of the right-handed state of the open chiral molecule in the aqueous bath at fixed tunneling frequency *ω*_*x*_ = 10^−3^ for *ω*_*z*_ = 10^−5^ (blue), *ω*_*z*_ = 10^−4^ (orange) and *ω*_*z*_ = 10^−3^ (green). The initial state is the left-handed one. Note that all parameters are made dimension-less with respect to the relevant characteristic parameters of the molecule. The figure is updated from ref. 47.

First we should specify all possible initial states of the odorant. We assume that the odorant is approximately isolated outside the nasal cavity. The energy splitting of the odorant is similar to the mean thermal energy (see Physical Parameters subsection, Results section). Therefore, the odorant is initially prepared in a superposition of ground and first excited states, |*E*_1_〉 and |*E*_2_〉, or equivalently a superposition of left- and right-handed states, |*L*〉 and |*R*〉. In a double-well potential, the energy states are delocalized over both wells, while the handed states are localized in the respective wells. In molecules, such handed states are corresponding to well-defined molecular structures. In chiral molecules, these structures are non-superposable to each other. The interaction of the molecule with a dilute environment leads to a incoherent mixture of left- and right-handed states (see ref. 47 and references therein). This is primarily due to the positional structure of the interaction Hamiltonian between the molecule and the surrounding environment. The molecule in interaction with a condensed environment (like a biological bath), can preserve the initial state. This phenomenon is known as quantum Zeno effect and is observed in a variety of biological systems (see e.g. refs 48 and 49). This effect is a consequence of the continues strong collisions of the condensed environment with the system. For a molecule confined in the double-well potential, we have shown that if the chiral interactions induced by a condensed biological environment are strong enough they can suppress the tunneling process and freeze the molecule in its initial state (see Fig. 7)^40, 47^.

Therefore, the odorant’s initial state before entering the ORs can be considered as a pure state. We consider the odorant’s initial state as the eigenstates of the relevant part of (13), namely the left- and right-handed states. However, in order to complete the picture we consider the ground state of energy as another initial state to include all the transitions that may be possible for the odorant.

### Polaron Transformation

The unperturbed Hamiltonian ${\hat{H}}_{0}={\hat{H}}_{od}+{\hat{H}}_{e}+{\hat{H}}_{env}$ is diagonalized by a polaron transformation as ref. 23

10 $${\hat{H}}_{0}^{^{\prime} }=\sum _{R=A,D}\,({\epsilon }_{R}+{\eta }_{R}{\hat{\sigma }}_{z})|R\rangle \langle R|+\sum _{i}\,{\omega }_{i}{\hat{b}}_{i}^{\dagger }{\hat{b}}_{i}$$

where

11 $$\begin{array}{rcl}{\eta }_{A} & = & -\frac{{\omega }_{z}}{2}\,\cos \,\{{\tan }^{-1}\,(\frac{{\omega }_{x}+{\gamma }_{A}}{{\omega }_{z}})\}\\ {\eta }_{D} & = & -\frac{{\omega }_{z}}{2}\,\cos \,\{{\tan }^{-1}\,(\frac{{\omega }_{x}-{\gamma }_{D}}{{\omega }_{z}})\}\end{array}$$

Similarly, the interaction Hamiltonian is transformed to

12 $$\begin{array}{rcl}{\hat{H}}_{{int}}^{^{\prime} } & = & {\rm{\Delta }}|A\rangle \langle D|\,\exp \,\{\frac{i}{2}[{\tan }^{-1}\,(\frac{{\omega }_{x}+{\gamma }_{A}}{{\omega }_{z}})+{\tan }^{-1}\,(\frac{{\omega }_{x}-{\gamma }_{D}}{{\omega }_{z}})]{\hat{\sigma }}_{y}\})\\  &  & \times \,\exp \,\{\sum _{i}\,(\frac{{\gamma }_{i,D}-{\gamma }_{i,A}}{{\omega }_{i}})\,({\hat{b}}_{i}^{\dagger }-{\hat{b}}_{i})\}+h.c\end{array}$$

#### Dynamics

Time evolution operator in the interaction picture at weak-coupling limit can be written as

13 $${\hat{U}}_{I}(t)=1-i{\int }_{0}^{t}\,d{t}_{1}{\hat{H}}_{int}({t}_{1})-{\int }_{0}^{t}\,d{t}_{1}^{^{\prime} }{\int }_{0}^{{t}_{1}^{^{\prime} }}\,d{t}_{1}{\hat{H}}_{int}({t}_{1}^{^{\prime} }){\hat{H}}_{int}({t}_{1})$$

We assume that initially the electron is located at the donor site |*D*〉, the odorant is found in the ground state of energy or left- or right-handed states (see Fig. 1), all denoted by |*i*〉, and the environment is described by the density matrix *ρ*_*env*_(0). The initial state of the whole system is then $\rho \mathrm{(0)}=|D,i\rangle \langle D,i|{\rho }_{env}\mathrm{(0)}$. The density matrix of the whole system at time *t* is given by ${\rho }_{I}(t)={\hat{U}}_{I}(t)\rho \mathrm{(0)}{\hat{U}}_{I}^{\dagger }(t)$. The probability of finding the electron at time *t* on the acceptor site and the odorant in the state |*j*〉 is obtained as

14 $$\begin{array}{rcl}{P}{{r}}_{D,i\to A,j} & = & T{r}_{env}\langle A,j|{\hat{U}}_{I}(t)\rho (0){\hat{U}}_{I}^{\dagger }(t)|A,j\rangle \\  & = & {{\rm{\Delta }}}^{2}{\int }_{0}^{t}\,d{t}_{1}^{^{\prime} }{\int }_{0}^{{t}_{1}^{^{\prime} }}\,d{t}_{1}{e}^{-i\epsilon ({t}_{1}-{t}_{1}^{^{\prime} })}\,\langle j|\hat{{\rm{\Omega }}}({t}_{1})|i\rangle \langle i|\hat{{\rm{\Omega }}}({t}_{1}^{^{\prime} })|j\rangle f(\omega ,{t}_{1},{t}_{1}^{^{\prime} })\end{array}$$

where $\hat{{\rm{\Omega }}}(t)$ is a matrix with elements

15 $$\begin{array}{l}{{\rm{\Omega }}}_{11}(t)={{\rm{\Omega }}}_{22}(-t)=\,\cos \,\{\frac{1}{2}[{\tan }^{-1}\,(\frac{{\omega }_{x}+{\gamma }_{A}}{{\omega }_{z}})+{\tan }^{-1}\,(\frac{{\omega }_{x}-{\gamma }_{D}}{{\omega }_{z}})]\}{e}^{-it({\eta }_{2}-{\eta }_{1})}\\ {{\rm{\Omega }}}_{12}(t)=-{{\rm{\Omega }}}_{21}(-t)=\,\sin \,\{\frac{1}{2}[{\tan }^{-1}\,(\frac{{\omega }_{x}+{\gamma }_{A}}{{\omega }_{z}})+{\tan }^{-1}\,(\frac{{\omega }_{x}-{\gamma }_{D}}{{\omega }_{z}})]\}{e}^{-it({\eta }_{1}+{\eta }_{2})}\end{array}$$

and $f(\omega ,{t}_{1},{t}_{1}^{^{\prime} })$ is the correlation function of the environment, defined by

16 $$f(\omega ,{t}_{1},{t}_{1}^{^{\prime} })=T{r}_{env}\{\hat{{\rm{\Theta }}}({t}_{1}){\rho }_{env}\mathrm{(0)}{\hat{{\rm{\Theta }}}}^{\dagger }({t}_{1}^{^{\prime} })\}$$

where $\hat{{\rm{\Theta }}}(t)$ is a displacement operator

17 $$\hat{{\rm{\Theta }}}(t)=\exp \,\{\sum _{i}\,\frac{{\gamma }_{i,D}-{\gamma }_{i,A}}{{\omega }_{i}}({e}^{i{\omega }_{i}t}{\hat{b}}_{i}^{\dagger }-{e}^{-i{\omega }_{i}t}{\hat{b}}_{i})\}$$

To calculate the environmental correlation function (16) we should specify the initial state of the environment. Regarding the environment in the thermal equilibrium, the corresponding correlation function is obtained as

18 $$\begin{array}{rcl}f(\omega ,{t}_{1},{t}_{1}^{\text{'}}) & = & T{r}_{env}\{\hat{{\rm{\Theta }}}({t}_{1})(\frac{1}{1+\tilde{n}}\sum _{n=0}^{\infty }\,{(\frac{\tilde{n}}{1+\tilde{n}})}^{n}|n\rangle \langle n|)\,{\hat{{\rm{\Theta }}}}^{\dagger }({t}_{1}^{^{\prime} })\}\\  & = & {e}^{iIm\zeta ({t}_{1})\zeta ({t}_{1}^{^{\prime} })}\frac{1}{1+\tilde{n}}\sum _{n=0}^{\infty }\,{(\frac{\tilde{n}}{1+\tilde{n}})}^{n}\langle n|{e}^{{\sum }_{i}{\chi }_{i}{\hat{b}}_{i}^{\dagger }-{\chi }_{i}^{\ast }{\hat{b}}_{i}}|n\rangle \end{array}$$

where $\tilde{n}=\mathrm{1/(}{e}^{\omega /{k}_{B}T}-\mathrm{1)}$, |*n*〉 is the number state and ${\chi }_{i}:=\zeta ({t}_{1})+\zeta ({t}_{1}^{^{\prime} })$. To obtain a closed mathematical form for correlation function we use the following relation for spectral density *J*(*ω*) of the environmental particles

19 $$J(\omega )=\sum _{i}\,{({\gamma }_{i,D}-{\gamma }_{i,A})}^{2}\delta (\omega -{\omega }_{i})\equiv {J}_{0}\omega {e}^{-\frac{\omega }{\lambda }}$$

where *J*_0_ is a measure of the coupling between the system and environment, and *λ* is the cut-off frequency of the environmental particles. Inserting (19) in (18), correlation function is summed up as

20 $$f(\omega ,{t}_{1},{t}_{1}^{^{\prime} })=\exp \,\{-{\int }_{0}^{\infty }\,\frac{J(\omega )}{{\omega }^{2}}[1-\,\cos \,(\omega ({t}_{1}-{t}_{1}^{^{\prime} }))f(\omega )-i\,\sin \,(\omega ({t}_{1}-{t}_{1}^{^{\prime} }))]\}$$

where for the environment in the ground state *f*(*ω*) = 1 and for the thermal environment $f(\omega )=\,\coth \,(\omega \mathrm{/2}{k}_{B}T)$. Note that the all rates are evaluated by using the relation (14). If we integrate over *t*_1_ and ${t}_{1}^{^{\prime} }$, we arrive at a function of *t*. Since the biological environment has no memory (i.e. Markov approximation), we safely replace *t* with infinity.
